# Intravascular Imaging Versus Physiology Assessment for Intermediate Lesions During PCI

**DOI:** 10.3390/biomedicines14061397

**Published:** 2026-06-21

**Authors:** Marios Sagris, Athanasios Makris, Svetlana Aghayan, Stergios Soulaidopoulos, Alexios Giannakodimos, Konstantinos Platanias, Andreas Tzoumas, Nikolaos Ktenopoulos, Konstantinos Pamporis, Nikolaos Stalikas, Gerasimos Gavrielatos, Efstratios Karagiannidis, Nikolaos Patsourakos, Dimitris Tousoulis, Konstantinos Tsioufis

**Affiliations:** 1 School of Medicine, Hippokration General Hospital, National and Kapodistrian University of Athens, 106 79 Athens, Greece; thanosmak4655@gmail.com (A.M.); dr.svetlana.aghayan@gmail.com (S.A.); soulaidopoulos@gmail.com (S.S.); konstantinosplatanias99@gmail.com (K.P.); nikosktenop@gmail.com (N.K.); drtousoulis@hotmail.com (D.T.); ktsioufis@gmail.com (K.T.); 2 Department of Cardiology, “Tzaneio” General Hospital of Piraeus, 185 36 Piraeus, Greece; alexisgiannak@hotmail.com (A.G.); makisgab@yahoo.gr (G.G.); drpats@yahoo.gr (N.P.); 3 Division of Cardiovascular Health and Disease, University of Cincinnati Medical Center, Cincinnati, OH 45219, USA; andreastzoumas@hotmail.com; 4 Women’s Heart Center, The Christ Hospital Heart and Vascular Institute, Cincinnati, OH 45219, USA; 5 The Carl and Edyth Lindner Center for Research and Education, The Christ Hospital, Cincinnati, OH 45219, USA; 6 Department of Hygiene, Social-Preventive Medicine & Medical Statistics, Medical School, Aristotle University of Thessaloniki, 541 24 Thessaloniki, Greece; konstantinospab@gmail.com; 7 Cardiovascular Center Aalst, AZORG Hospital, 9300 Aalst, Belgium; nstalik@gmail.com; 8 Department of Emergency Medicine, AHEPA University Hospital, 54 636 Thessaloniki, Greece; stratoskarag@gmail.com

**Keywords:** PCI, intravascular imaging, IVUS, OCT, physiology, iFR, FFR, intermediate lesions

## Abstract

Coronary artery disease (CAD) is the leading cause of mortality, with percutaneous coronary intervention (PCI) constituting the gold standard treatment. However, in an important proportion of the cases, lesion severity is debatable since conventional angiography provides only a two-dimensional representation of the vessel lumen and fails to quantify ischemic significance or plaque morphology. In these cases, several adjunctive tools considering physiology and imaging guidance may assist in quantifying the severity of the disease. Imaging modalities such as intravascular ultrasonography (IVUS) and optical coherence tomography (OCT), as well as physiology such as fractional flow reserve (FFR) and instantaneous wave-free (iFR), revolutionized revascularization strategy by linking anatomical stenosis to its functional consequence on myocardial perfusion. The present review summarizes and contrasts the available evidence for physiology and imaging guidance, considering the assessment of intermediate lesions during PCI and providing insights for their use in specific lesion subsets.

## 1. Introduction

In patients with coronary artery disease (CAD), the degree of luminal narrowing, plaque characteristics and the physiologic significance of the lesion are prognostic indicators [1,2]. Conventional angiography provides only a two-dimensional representation of the vessel lumen and fails to quantify ischemic significance or plaque morphology. Imaging modalities such as intravascular ultrasonography (IVUS) and optical coherence tomography (OCT) are commonly used adjunctive techniques that can provide detailed anatomical information about the lumen, vessel, and plaque distribution. Both modalities can be used during the PCI procedure to aid in stent placement and minimize stent-related issues [3]. Several trials and meta-analyses presented improved clinical outcomes, with imaging-guided PCI compared with sole angiography-guided PCI [4,5,6,7]. Physiology, on the other hand, such as fractional flow reserve (FFR) and instantaneous wave-free (iFR), introduced as pressure-derived indexes of lesion-specific ischemia, revolutionized revascularization strategy by linking anatomical stenosis to its functional consequence on myocardial perfusion [8]. FFR is defined as the maximal blood flow to the myocardium in the presence of a stenosis in the supplying coronary vessel, divided by the theoretical normal maximal flow in the same distribution. This index constitutes the fraction of the normal maximal myocardial flow that can be achieved despite the coronary stenosis. The normal value of the index is 1.0, regardless of the patient or the specific vessel studied [9]. FFR values of less than 0.75 identify functionally important stenoses [10]. The same concept applies to iFR but without the need for hyperemia to induce ischemia. Numerous trials have shown that FFR- and iFR-guided PCI is associated with fewer clinical events than angiography-guided PCI and medical treatment [11,12,13,14,15].

Although the basic concepts underlying the use of physiology and imaging during PCI differ between each other, both are the most commonly used adjunctive tools in the diagnosis and treatment of CAD during cardiac catheterization. The present review summarizes and contrasts the available evidence for physiology and imaging guidance, focusing on head-to-head comparative studies, and discusses their respective significance across the spectrum of chronic and acute coronary syndromes. Understanding their relative advantages, limitations, and clinical contexts of each modality is essential for tailoring individualized revascularization strategies in the era of precision coronary intervention.

## 2. Invasive Physiological Assessment in Coronary Stenosis

Invasive physiological assessment has become a cornerstone in the evaluation and management of coronary artery disease (CAD), offering functional information that cannot be reliably obtained from angiography alone. The clinical rationale for performing physiological measurements during coronary catheterization is particularly strong in the context of intermediate lesions (~60–70% diameter stenosis), in which angiographic severity correlates poorly with hemodynamic significance. Physiological assessment enables identification of ischemia-producing lesions, reduces unnecessary revascularization, informs PCI strategy, and improves both procedural and long-term outcomes. Over the past two decades, indices such as fractional flow reserve (FFR), instantaneous wave-free ratio (iFR), and resting full-cycle ratio (RFR) have demonstrated substantial diagnostic and prognostic value, contributing significantly to contemporary revascularization practice [16,17,18,19,20,21,22,23,24].

FFR quantifies the ratio of maximal blood flow in a stenotic artery to the theoretical maximal flow if the vessel were normal. Achieving this requires pharmacologically induced hyperemia, most commonly using adenosine, to minimize microvascular resistance and maximize coronary flow [22,23,24]. Pressure-sensitive guidewires measure the distal coronary pressure (Pd) relative to aortic pressure (Pa), and FFR values ≤ 0.80 typically indicate hemodynamically significant stenosis [23,24]. Extensive evidence supports the prognostic and therapeutic utility of FFR-guided PCI. Randomized trials and meta-analyses demonstrate that FFR-guided revascularization reduces unnecessary stenting, lowers periprocedural complication rates, and improves long-term outcomes in stable CAD [22,23,24]. Beyond percutaneous treatment, FFR has also found utility in surgical planning for coronary artery bypass grafting (CABG), particularly for selecting optimal graft targets and improving long-term graft patency [21].

iFR was developed to overcome limitations associated with FFR, primarily the need for pharmacologic hyperemia. By measuring the pressure ratio during the diastolic wave-free period—when microvascular resistance is naturally minimized—iFR eliminates the need for vasodilators, reduces procedural time, and improves patient comfort [20]. Multiple studies have confirmed a strong correlation between iFR and FFR, although discordance may occur in patients with microvascular dysfunction or borderline lesions [17,18,19,20]. These characteristics have contributed to the widespread adoption of iFR in routine clinical use [16,17,18,19,20]. Hybrid strategies combining resting indices with FFR have further improved diagnostic accuracy and procedural efficiency, particularly in cases in which iFR results fall within a gray zone [25,26,27].

RFR represents another non-hyperemic physiological index, calculated as the lowest distal-to-aortic pressure ratio across the entire cardiac cycle [19,20]. Similar to iFR, RFR correlates strongly with FFR and provides an alternative assessment in patients for whom hyperemia is contraindicated [17,18,19,26]. Evidence suggests that RFR-guided revascularization yields outcomes comparable to those obtained with FFR; however, discrepancies may arise in the presence of microvascular dysfunction or in acute coronary syndromes (ACSs), necessitating careful interpretation [16,18,19,28]. Although RFR has shown good correlation with established physiological indices such as FFR and iFR, its evidence base remains comparatively limited, particularly regarding randomized clinical outcome data. This limitation is even more pronounced in ACSs, where the influence of microvascular dysfunction further restricts validation, and therefore RFR should not yet be considered equivalent to FFR or iFR in terms of clinical evidence and guideline-supported application.

### 2.1. Comparative Evidence for Intermediate Lesions Based on Physiology

In CCS, extensive evidence confirms that FFR-guided PCI is superior to angiography-guided management for reducing major adverse cardiovascular events (MACE), unnecessary stent placement, and long-term complications [22,23,24]. In the specific context of intermediate stenoses (~60–70%), FFR provides reliable discrimination between ischemia-producing and non-flow-limiting lesions. Several comparative studies have demonstrated that non-hyperemic indices, including iFR and RFR, achieve similar diagnostic performance in stable disease settings [16,17,20,26].

Additional studies such as the FiGARO trial [26] have examined sources of discordance between FFR and iFR and found that diffuse disease and microvascular dysfunction are primary contributors—situations highly relevant for borderline lesions [27]. Beyond this, large randomized trials have confirmed the long-term safety and efficacy of non-hyperemic indices in CCS. The iFR-SWEDEHEART trial was a multicenter, randomized study that compared iFR with fractional flow reserve (FFR) for guiding coronary revascularization in patients with stable angina or acute coronary syndromes. A total of 2037 patients with intermediate coronary stenoses were randomized to iFR- or FFR-guided PCI, using cut-off values of ≤0.89 for iFR and ≤0.80 for FFR; it was demonstrated that iFR-guided PCI was non-inferior to FFR-guided PCI for the composite endpoint of MACE over a 5-year follow-up, with similar rates of death, myocardial infarction, and repeat revascularization. Similarly, the DEFINE-FLAIR trial analyzed a total of 2492 patients with intermediate coronary lesions who were randomized to iFR- or FFR-guided strategies. It showed that iFR-based decision-making led to comparable long-term clinical outcomes to FFR-guided strategies, while additionally reducing the need for adenosine administration and procedural time [29,30]. These data support a pragmatic approach in CCS: non-hyperemic indices may be used for initial assessment in isolated intermediate lesions, with hyperemic confirmation recommended for gray-zone or anatomically complex lesions [25,26,27]. Limitations of the current evidence include methodological heterogeneity, inconsistent definitions of intermediate stenosis, and variable operator thresholds for physiological testing, all of which should be considered when interpreting study findings.

In ACS, physiological assessment poses specific challenges related to dynamic microvascular dysfunction, inflammation, and transient vasomotor disturbances, all of which may alter pressure-derived indices [18,19,28]. Although FFR remains the reference standard in ACS for assessing non-culprit lesions, early measurements may be affected by microvascular obstruction. Resting indices such as iFR and RFR may underestimate lesion severity in ACS conditions, contributing to higher rates of discordance with FFR [18,19,28]. Studies including Trøan et al. [28] have demonstrated that microcirculatory impairment significantly affects RFR measurements, reinforcing the need for caution. Additionally, studies indicate that microvascular “stunning” immediately post-ACS can transiently reduce hyperemic response, limiting FFR reliability and causing dynamic changes in coronary flow and microvascular resistance over the first 24 h post-PCI [31]. Comparative analyses consistently show that non-hyperemic indices have reduced reliability immediately following ACS, particularly for intermediate (~60–70%) lesions. Consequently, when resting indices yield borderline or discordant results, confirmatory hyperemic assessment is recommended, or physiological evaluation may be deferred until microvascular recovery if clinically acceptable [18,19,28].

### 2.2. Limitations of Physiological Indices

Despite their demonstrated value, all physiological indices have inherent limitations. FFR requires administration of hyperemic agents, which may cause discomfort and are contraindicated in patients with severe asthma, hypotension, or conduction disturbances [22,23]. Resting indices (iFR and RFR) avoid hyperemia but may be influenced by heart rate variability, diffuse atherosclerosis, or microvascular dysfunction, leading to occasional discordance with FFR [16,18,19]. Technical variables such as wire drift, calibration errors, and operator experience also affect accuracy [22,25]. Complex lesions, including left main stenosis, tandem lesions, and diffuse disease, remain challenging for pressure-based methods, reinforcing the need for individualized assessment strategies [19,22,32]. A hybrid diagnostic model—using resting indices for initial screening and FFR for borderline or complex lesions—is increasingly favored and supported by clinical evidence [25,26,27]. FFR remains particularly advantageous in complex anatomy, gray-zone resting-index values, and ACS with suspected microvascular dysfunction [17,19,26,28].

## 3. Intravascular Imaging (IVUS and OCT)

Intravascular imaging complements physiological assessment by providing high-resolution structural evaluation of the coronary arterial wall, plaque morphology, and stent–vessel interactions. IVUS and OCT share the objective of improving procedural planning and outcomes but differ notably in their imaging principles, resolution, and penetration capabilities.

IVUS employs high-frequency ultrasound (20–60 MHz) to provide 360° vessel wall visualization with axial resolution of 80–150 µm and penetration of 4–8 mm [33]. This allows comprehensive assessment of vessel dimensions, external elastic membrane boundaries, plaque burden, remodeling patterns, and detection of calcified or fibrotic lesions that may influence stent expansion. IVUS can assess minimal lumen area (MLA) and plaque composition, facilitating evidence-based decisions regarding the need for revascularization in intermediate lesions. IVUS is particularly valuable for assessing complex coronary anatomies, including bifurcation lesions, left main disease, and diffuse atherosclerosis, as it allows accurate sizing of stents, optimization of deployment, and reduction in malapposition. Additionally, it provides guidance in evaluating stent under-expansion, edge dissections, and late stent complications, which are major contributors to restenosis and stent thrombosis [33,34,35].

OCT uses near-infrared light to achieve superior axial resolution (10–20 µm), though penetration is limited to 1–2 mm and requires transient displacement of blood with contrast or saline [4,36]. OCT excels in characterizing superficial plaque components, including thin-cap fibroatheromas, lipid-rich cores, calcification patterns, macrophage infiltrates, thrombus, microchannels, and neovascularization [37,38]. OCT is particularly useful in acute coronary syndromes (ACSs), allowing precise identification of plaque rupture, erosion, or calcified nodules, which can inform timing and strategy for PCI. OCT also enables high-resolution assessment of stent apposition, tissue prolapse, strut coverage, and thrombus burden, facilitating immediate intraprocedural optimization and reducing the risk of adverse outcomes [37,39,40]. Moreover, it is essential in the evaluation of non-culprit lesions in cases of multivessel disease. By integrating detailed morphological information with clinical and procedural data, OCT may support increasingly personalized post-infarction management, including tailored antithrombotic, lipid-lowering, and anti-inflammatory therapies [41].

Randomized trials such as IVUS-XPL and ULTIMATE have demonstrated that IVUS-guided PCI significantly reduces MACE, stent thrombosis, and target vessel revascularization compared with angiography-guided PCI [4,34,36]. Large meta-analyses including more than 18,000 patients confirm reductions in all-cause mortality, cardiac death, MACE, and repeat revascularization with imaging-guided PCI [33]. OCT-guided trials, including ILUMIEN I–III and OCTIVUS, have shown improved stent optimization, reduced procedural complications, and outcomes comparable or superior to IVUS, particularly in ACS and complex lesion subsets [39,40,42]. Moreover, OCT provides mechanistic insight into lesion morphology that explains physiological discordance between FFR/iFR and anatomical severity, aiding in more precise, patient-tailored intervention strategies. IVUS and OCT are most effective when used in conjunction with physiological indices (FFR, iFR, and RFR). Imaging can clarify ambiguous functional measurements, detect focal versus diffuse disease, and guide stent sizing and optimization in lesions that demonstrate intermediate or borderline physiological significance. Hybrid approaches—using initial non-hyperemic or hyperemic physiological assessment followed by intravascular imaging for complex or gray-zone lesions—have been shown to improve procedural outcomes, reduce adverse events, and provide both mechanistic and prognostic information in real-world cohorts [4,34,35,36,37,38,43,44].

### 3.1. Comparative Evidence for Intermediate Lesions Based on Imaging

In intermediate lesions, intravascular imaging offers diagnostic and therapeutic advantages that complement physiological measurements. Imaging assists in distinguishing anatomic features and clarifies the true significance of coronary lesions in both chronic coronary syndrome (CCS) and acute coronary syndrome (ACS). In CCS, IVUS and OCT improve the assessment of lesion severity by identifying positive remodeling, plaque burden, and complex morphology that may appear severe angiographically but are not necessarily hemodynamically significant. In ACS, intravascular imaging additionally characterizes plaque pathology—such as rupture, erosion, calcified nodules, or thrombotic components—which can influence both the indication for PCI and the choice of interventional strategy. IVUS quantifies vessel dimensions and plaque burden, whereas OCT provides detailed plaque phenotyping, including fibrous, lipid-rich, calcified, or thrombotic features [29,32,35,36,40]. The use of intravascular imaging can also provide important insights in otherwise difficult cases where physiology is lagging. More particularly, it can identify cases of diffuse disease, significant calcification, or large luminal dimensions and explain discrepancies between resting indices and FFR. The FORZA trial [44] and the study by Yang et al. [43] highlight the complementary roles of physiology and intravascular imaging in the assessment of intermediate coronary stenoses. FORZA demonstrated that FFR-based guidance safely reduces the need for revascularization, whereas OCT-guided management leads to more frequent PCI and improved symptom control. In contrast, the large-scale analysis by Yang et al. showed comparable clinical outcomes between FFR- and IVUS-guided strategies while identifying IVUS-derived anatomical thresholds (e.g., MLA and plaque burden) that refine risk stratification for both deferred and treated lesions. Together, these findings support an integrated approach in which physiological assessment guides the decision to revascularize, and intravascular imaging optimizes lesion characterization and procedural results. Intravascular imaging may also assist the operator and guide the stenting technique. IVUS-guided stent sizing and expansion correlate with improved outcomes, as demonstrated in large-scale trials and criteria-evaluation studies [18,43]. OCT identifies malapposition, tissue prolapse, edge dissections, and thrombus burden, enabling immediate corrections that improve prognosis [37,39,40,42].

Considering ACS cases, OCT studies reveal the presence of plaque rupture in 59%, plaque erosion in 32%, and calcified nodules in 9% of culprit lesions [37]. These observations underscore the central role of intravascular imaging in defining the underlying mechanism of ACS, as angiography alone cannot reliably differentiate between rupture, erosion, or calcified nodules. Importantly, this mechanistic insight extends beyond the culprit lesion: in patients with ACS, intermediate non-culprit plaques frequently exhibit complex morphology, including large lipid pools, thin-cap fibroatheromas, or significant plaque burden, even in the absence of hemodynamically significant stenosis. Because microvascular dysfunction, diffuse disease, and dynamic changes in coronary tone can render physiological assessment unreliable in the acute setting, OCT- or IVUS-based characterization of plaque phenotype provides additional information that may influence the timing, strategy, and necessity of revascularization. Identifying high-risk morphological features in non-culprit lesions—such as thin fibrous caps, large lipid cores, macrophage accumulation, or microcalcifications—may help guide closer surveillance or staged intervention, whereas stable-appearing plaques (e.g., fibrotic or thick-cap lesions) may safely be managed conservatively. Thus, in ACS, plaque morphology assessment complements physiology and offers a more comprehensive approach to risk stratification and decision-making for intermediate lesions. This mechanistic insight is particularly relevant for intermediate non-culprit plaques, where physiology may be unreliable and plaque morphology may guide timing and need for revascularization [45,46,47,48,49].

Recent meta-analyses suggest that a more aggressive imaging-guided PCI strategy improves long-term outcomes without compromising procedural safety. An updated meta-analysis of 24 randomized trials including 14,037 patients demonstrated that intravascular imaging-guided PCI reduced MACE, MI, target vessel revascularization, and stent thrombosis compared with angiography-guided PCI [50]. Furthermore, a 2024 meta-analysis of OCT-guided PCI reported lower rates of cardiovascular death, stent thrombosis, and MACE despite more frequent procedural optimization maneuvers. These findings suggest that the additional interventions prompted by intravascular imaging may translate into improved long-term efficacy without an apparent increase in periprocedural complications [51].

### 3.2. Limitations of Imaging Indices

Given the context in which they are used, each technology has unique strengths and limitations. IVUS can provide excellent penetration for larger vessels but has lower microstructural resolution than OCT, making subtle plaque characterization less precise. OCT requires contrast injection, which may be limited in patients with renal insufficiency, and has reduced penetration in heavily calcified or large vessels. Operator experience remains a key determinant of image interpretation and clinical impact. Imaging is particularly advantageous in scenarios where angiography is insufficient, including long or complex lesions, left main and bifurcation disease, heavily calcified vessels, ACS mechanisms, and the evaluation of stent failure [4,34,35,36,43,52]. Combining physiological assessment with intravascular imaging yields the most comprehensive evaluation and supports truly patient-specific revascularization planning.

For angiographically intermediate coronary stenoses (~60–70%), a combined approach using physiological indices (FFR, iFR, and RFR) and intravascular imaging (IVUS and OCT) offers the most robust framework for clinical decision-making. FFR remains the gold standard, particularly in ACS, complex anatomy, or gray-zone resting-index scenarios. Non-hyperemic indices provide practical alternatives in stable patients and for initial triage. Intravascular imaging refines anatomic and morphological understanding, clarifies physiological discordance, and optimizes PCI outcomes. By providing detailed anatomical, morphological, and procedural guidance, IVUS and OCT enhance the precision of PCI, improve patient outcomes, and help reconcile discrepancies between functional and structural assessment. An individualized, evidence-based strategy integrating both functional and structural assessments ensures precise lesion evaluation, minimizes unnecessary interventions, and improves long-term cardiovascular outcomes.

## 4. Head-to-Head Comparisons

The comparative evidence between physiology-guided and imaging-guided PCI reflects two different philosophies of coronary assessment. Physiology identifies ischemia, whereas intravascular imaging characterizes plaque morphology and optimizes PCI. Although traditionally viewed as alternative strategies, the most robust evidence suggests that they are complementary to each other. PCI of intermediate size lesions has certainly undergone a paradigm shift in recent years as angiography alone frequently misclassifies intermediate lesions [13]. Specifically, on the one hand, up to 40% of angiographically intermediate lesions are not ischemic by FFR or iFR, and on the other hand, IVUS demonstrates that many stenoses judged “moderate” angiographically have large plaque burdens (>70%) indicative of high-risk morphology [13,14]. Thus, this sets the stage for comparative evaluation as each tool examines different dimensions of the same lesion.

Emerging evidence with a few studies in the past decade that compared FFR- and IVUS-guided coronary intervention strategies in patients with de novo coronary intermediate lesions is gaining attention. The very first direct comparison of physiology versus imaging-guided PCI was reported by Nam et al. back in 2010 with a comparison of FFR and IVUS [53]. It was a single-center, prospective cohort of 167 patients with intermediate lesions. The major findings in this study can be summarized as follows: (1) FFR- or IVUS-guided PCI in patients with intermediate coronary lesions was associated with favorable clinical outcomes; and (2) the rate of performed PCI in intermediate coronary lesions was much lower in the FFR-guided compared with the IVUS-guided group without any increase in adverse event rates according to established criteria [53]. The latter emphasizes a potential over-treatment tendency when IVUS is used as the primary gatekeeper [54].

The FLAVOUR trial represents the largest and most definitive head-to-head comparison to date. This multicenter RCT enrolled 1682 patients with angiographically intermediate stenoses (40–70%) and randomized them to FFR-guided or IVUS-guided PCI strategies [55]. At the 24-month follow-up, no significant difference was observed in hard clinical outcomes between FFR-guided and IVUS-guided PCI. PCI was performed significantly less often in the FFR group [55], confirming the already existing evidence [44,53,56]. Overall, the FLAVOUR trial emphasized the clinical non-inferiority of FFR guidance when compared to IVUS, as well as its role in deferring unnecessary interventions. The FLAVOUR II trial extended this comparison to involve angiography-derived FFR (AngioFFR), a specific wire-free physiology index derived from coronary angiography [57]. Approximately 1800 patients were randomized to AngioFFR-guided or IVUS-guided strategies. The primary endpoint (death, MI, or revascularization at 12 months) was similar between groups, with AngioFFR again yielding fewer PCIs and shorter procedural times. The study reinforced the fact that noninvasive physiology can match imaging in safety and efficacy in relatively stable patients with angiographically intermediate stenoses.

However, both studies revealed important nuances. First, in non-ischemic lesions, IVUS sometimes recommended PCI due to large plaque burden or ambiguous lumen loss, whereas in physiologically positive lesions, the IVUS-derived external elastic membrane (EEM) diameter frequently upstaged stent size compared to angiography or physiology-guided sizing. Thus, the existing comparative evidence of imaging vs. physiology-guided PCI suggests a strategic divergence rather than a superiority contest.

As to comparisons between OCT and FFR, robust data from large multicenter trials are lacking. However, in a single-center randomized study, the use of OCT was safe, causing a larger number of PCIs, but was also associated with a lower occurrence of the combined endpoint of MACE and significant angina, whereas FFR was associated with a higher rate of medically treated patients and lower costs. The conservative approach of patients in this trial with angiographically intermediate stenoses when using FFR guidance showed a benefit in terms of renal preservation [44].

Both physiological and intravascular imaging techniques rely on operator dependence, requiring expertise for image acquisition, interpretation, and integration into clinical decision-making. Their widespread adoption is also constrained by additional procedural costs, variable reimbursement policies, and limited availability of specialized equipment across healthcare systems. Furthermore, dedicated training and maintenance of procedural proficiency are essential to ensure optimal use of these technologies, highlighting the need for standardized education programs and broader accessibility.

## 5. Physiological vs. Imaging Guidance in Specific Lesion Subsets

In specific settings such as LM disease where FFR/iFR accuracy is influenced by downstream disease, imaging techniques and especially IVUS can be helpful. Although IVUS is a poor determinant of whether a stenosis requires PCI, in the case of left main coronary artery, its role is well established [58]. In bifurcation lesions where uncertainty about performing an intervention rises, physiology determines which side branches are truly ischemic [59], with imaging techniques contributing to carina shift visualization, stent malapposition, and ostial plaque identification. In an analogous fashion, imaging techniques and mainly OCT with its high-resolution capacities can characterize calcific lesions as they can penetrate calcium, evaluate calcium thickness, and identify the severity and pattern of calcium requiring additional lesion modification [60]. In diffuse stenoses throughout the entire epicardial vessels, in contrast, physiology shows superiority as it distinguishes focal vs. diffuse patterns of ischemia [61].

Thus, current practice supports combining both approaches, with physiology determining whether a lesion requires intervention and subsequently imaging tools optimizing the procedure. This integrated strategy reduces stent use while improving long-term outcomes—emerging as the standard for precision PCI. Physiological indices such as FFR, iFR, and RFR determine whether an intermediate stenosis is functionally significant and likely to benefit from revascularization, thereby preventing unnecessary interventions in lesions that do not cause ischemia. Intravascular imaging, particularly IVUS and OCT, provides detailed anatomical information regarding plaque burden, vessel dimensions, calcium distribution, and stent optimization, allowing operators to tailor PCI strategy and minimize procedural complications (Table 1).

Importantly, the relationship between ischemia and plaque vulnerability is complex and often discordant. Not all ischemia-producing lesions exhibit high-risk plaque characteristics, while some non-flow-limiting lesions may harbor features associated with future adverse events, including large plaque burden, positive remodeling, thin-cap fibroatheroma, lipid-rich plaque, or macrophage infiltration [62]. Landmark studies such as PROSPECT, COMBINE OCT-FFR, and PREVENT have demonstrated that vulnerable plaque morphology provides prognostic information beyond physiological assessment alone [62]. Consequently, physiology identifies lesions requiring treatment based on their hemodynamic significance, whereas imaging enables the characterization of the underlying substrate and the recognition of plaques at increased risk of future instability.

**Table 1:** Summary of the available evidence considering physiology and imaging devices.

Modality	Type	Principle	Strengths	Limitations	Optimal Use	Key Cut-Offs
IVUS	Imaging	Ultrasound-based cross-sectional vessel imaging	Deep vessel penetration; accurate vessel sizing; assessment of plaque burden and remodeling; useful in left main disease	Lower spatial resolution vs. OCT; limited thrombus characterization	Stent sizing/optimization; left main and large vessels; ambiguous lesions	MLA (LM: ~6 mm^2^; non-LM: ~3–4 mm^2^ *) [63]
OCT	Imaging	Near-infrared light for high-resolution imaging	High spatial resolution; detailed plaque characterization (rupture, erosion, TCFA); thrombus detection	Limited penetration depth; requires contrast; less suitable in large vessels	ACS (culprit characterization); stent optimization; erosion vs. rupture	No universal cut-off; MLA ~1.5–2.0 mm^2^ [64]
FFR	Physiology	Hyperemic pressure ratio (distal/aortic pressure)	Gold standard for ischemia; robust evidence; guides revascularization	Requires hyperemia; affected in ACS (microvascular dysfunction); patient discomfort	CCS; non-culprit lesions in ACS	≤0.80 [65]
iFR	Physiology	Resting pressure ratio during wave-free period	No hyperemia; faster and patient-friendly; comparable outcomes to FFR	Borderline zone; affected by hemodynamics	CCS; non-culprit lesions in ACS	≤0.89 [14]
RFR	Physiology	Whole-cycle resting pressure ratio	No hyperemia; operator-independent; correlates with iFR/FFR	Less validated vs. FFR/iFR; limited outcome data	CCS; emerging use in ACS	≤0.89 [7]
QFR	Angiography-derived Physiology	Computational flow ratio from 3D angiography	No pressure wire; no hyperemia; fast	Dependent on image quality; limited in complex lesions/ACS	CCS; emerging tool for intermediate lesions	≤0.80 [66]
OCT-derived FFR	Hybrid (Imaging + Physiology)	Flow estimation based on OCT geometry	Combines anatomy and physiology; no pressure wire	Limited availability; evolving validation	Research/selected cases	~≤0.80 [67]
AI-IVUS	Imaging + AI	Automated plaque and vessel analysis	Operator-independent; reproducibility; plaque quantification	Limited availability; requires validation	Research; future clinical workflows	N/A
NIRS-IVUS	Hybrid Imaging	Lipid-core detection + IVUS structure	Identifies vulnerable plaques; lipid burden quantification	Limited availability; not functional assessment	Risk stratification; research	LCBI (≥400 maxLCBI4mm) [68] **
PPG (Pullback Pressure Gradient)	Physiology	Gradient distribution along vessel	Differentiates focal vs. diffuse disease; guides PCI strategy	Requires expertise; not widely adopted	CCS; complex lesions	No universal cut-off yet [69]

Abbreviations: ACS = acute coronary syndromes; CCS = chronic coronary syndrome; IVUS = intravascular ultrasound; OCT = optical coherence tomography; FFR = fractional flow reserve; iFR = instantaneous wave-free ratio; RFR = resting full-cycle ratio; QFR = quantitative flow ratio; NIRS = near-infrared spectroscopy; PPG = pullback pressure gradient; MLA = minimal lumen area; LCBI = lipid core burden index. * MLA thresholds vary by vessel size and population. ** maxLCBI4mm ≥ 400 associated with high-risk plaques.

## 6. Future Directions

Future directions in the assessment of intermediate coronary lesions increasingly focus on hybrid strategies that integrate intravascular imaging with physiological surrogates to provide a more comprehensive evaluation of lesion significance. Imaging-derived physiological indices, such as OCT-derived FFR and IVUS-based computational FFR, allow the estimation of lesion-specific ischemia using high-resolution anatomical data without the need for pressure wires or hyperemic agents [70]. Angiography-based techniques, including quantitative flow ratio (QFR) and its microvascular-adjusted iteration (μQFR), further simplify physiological assessment by enabling rapid, wire-free estimation of functional severity directly from routine angiographic images [71]. These approaches, particularly when combined with co-registration of imaging and angiography, have the potential to streamline PCI workflows while maintaining diagnostic accuracy, especially in patients with intermediate lesions or contraindications to hyperemia.

Concurrently, advances in intravascular imaging and pressure-based analytics are enhancing the understanding of lesion behavior beyond simple stenosis severity. Artificial intelligence-driven IVUS analysis (AI-IVUS) enables automated plaque characterization, vessel sizing, and outcome prediction, reducing operator variability and improving reproducibility. Near-infrared spectroscopy–IVUS (NIRS-IVUS) adds complementary information by identifying lipid-rich plaques, which may influence decision-making in lesions with borderline physiological significance [72,73]. Recent advancements in the diagnosis of MI with non-obstructive coronary arteries (MINOCA) highlight that although OCT remains the preferred modality for detecting subtle abnormalities, IVUS combined with NIRS may serve as a valuable alternative for identifying plaque rupture and erosion, particularly when OCT is not feasible. By providing complementary structural and compositional plaque assessment, the IVUS-NIRS approach may enhance the diagnostic evaluation of type 1 MINOCA and its underlying mechanisms [74]. In addition, novel physiological metrics such as the Pullback Pressure Gradient (PPG) provide insight into the spatial distribution of pressure loss along the vessel, helping to distinguish focal from diffuse disease and refine revascularization strategies [75]. As these technologies continue to evolve, their combined application may shift PCI guidance toward a precision-based paradigm that integrates anatomy, physiology, and plaque biology, with future randomized studies needed to define their optimal clinical implementation across both chronic and acute coronary syndromes.

## 7. Conclusions

In contemporary interventional cardiology, intravascular imaging and physiological indices should no longer be viewed as competing modalities, but rather as complementary tools providing distinct and synergistic information. Physiological assessment identifies the functional significance of intermediate lesions, whereas imaging characterizes plaque morphology, vulnerability, and procedural optimization. Experience and emerging evidence suggest that vulnerable plaques may not always produce ischemia, while functionally significant lesions may lack high-risk morphological features, highlighting the limitations of relying on a single modality alone. Therefore, the integration of both physiology and imaging is essential for comprehensive lesion assessment and for guiding individualized revascularization strategies in both CCS and ACS.

## Data Availability

No new data were created or analyzed in this study.
